# Optimization of proximity ligation assay (PLA) for detection of protein interactions and fusion proteins in non-adherent cells: application to pre-B lymphocytes

**DOI:** 10.1186/s13039-017-0328-2

**Published:** 2017-07-20

**Authors:** Lydie Debaize, Hélène Jakobczyk, Anne-Gaëlle Rio, Virginie Gandemer, Marie-Bérengère Troadec

**Affiliations:** 1 Institut de Génétique et Développement de Rennes, UMR 6290 CNRS, Université de Rennes 1, UBL, 2 avenue du Professeur Léon Bernard, 35043 RENNES CEDEX, France; 2 SFR Biosit UMS CNRS 3480/US INSERM 018, Rennes, France; 3 Centre Hospitalier Universitaire, Rennes, France

**Keywords:** Fusion gene, Fusion protein, PLA, Proximity ligation assay, Chromosomal rearrangement, B-cells, Non-adherent cells

## Abstract

**Background:**

Genetic abnormalities, including chromosomal translocations, are described for many hematological malignancies. From the clinical perspective, detection of chromosomal abnormalities is relevant not only for diagnostic and treatment purposes but also for prognostic risk assessment. From the translational research perspective, the identification of fusion proteins and protein interactions has allowed crucial breakthroughs in understanding the pathogenesis of malignancies and consequently major achievements in targeted therapy.

**Methods:**

We describe the optimization of the Proximity Ligation Assay (PLA) to ascertain the presence of fusion proteins, and protein interactions in non-adherent pre-B cells. PLA is an innovative method of protein-protein colocalization detection by molecular biology that combines the advantages of microscopy with the advantages of molecular biology precision, enabling detection of protein proximity theoretically ranging from 0 to 40 nm.

**Results:**

We propose an optimized PLA procedure. We overcome the issue of maintaining non-adherent hematological cells by traditional cytocentrifugation and optimized buffers, by changing incubation times, and modifying washing steps. Further, we provide convincing negative and positive controls, and demonstrate that optimized PLA procedure is sensitive to total protein level. The optimized PLA procedure allows the detection of fusion proteins and protein interactions on non-adherent cells.

**Conclusion:**

The optimized PLA procedure described here can be readily applied to various non-adherent hematological cells, from cell lines to patients’ cells. The optimized PLA protocol enables detection of fusion proteins and their subcellular expression, and protein interactions in non-adherent cells. Therefore, the optimized PLA protocol provides a new tool that can be adopted in a wide range of applications in the biological field.

**Electronic supplementary material:**

The online version of this article (doi:10.1186/s13039-017-0328-2) contains supplementary material, which is available to authorized users.

## Background

The vast majority of human hematological malignancies are caused by the clonal expansion of a single cell that has acquired genomic aberrations. Tumor-specific chromosomal translocations are frequent and contribute directly to malignant transformation. Such translocations, and other genetic abnormalities, have been described for many hematological malignancies, including acute or chronic lymphoid and myeloid leukemia, other myeloproliferative disorders, myelodysplastic syndromes, multiple myeloma, and malignant lymphomas.

From the clinical perspective, detection of chromosomal abnormalities in most hematological malignancies is relevant not only for diagnostic and treatment purposes but also for prognostic risk assessment. A description of chromosomal abnormalities is included in the 2008 World Health Organization Classification of Tumors of Hematopoietic and Lymphoid Tissues [1] and guides the practitioner in choosing the most appropriate treatment. Furthermore, chromosomal abnormalities identification harbors a prognosis value; for example patients bearing the chromosomal translocation t(12;21)(p13;q22) generating *ETV6-RUNX1* fusion gene have a better prognosis than those displaying chromosomal rearrangements in *MLL* gene [2, 3].

From a translational research perspective, the step from genetic identification of a chromosomal translocation to confirmation of the presence of the corresponding fusion protein has allowed crucial breakthroughs in understanding the pathogenesis of malignancies and consequently major achievements in targeted therapy. Historically, identification of the *BCR* and *ABL* genes involved in a balanced translocation between chromosomes 9 and 22 has led to the discovery of the BCR-ABL1 fusion protein, a constitutively active tyrosine kinase. From this discovery, other protein tyrosine kinase inhibitors, which are effective not only against the BCR-ABL1 fusion protein but also against other neoplasms producing protein tyrosine kinases, have been developed [4].

Current cytogenetic analyses are based on DNA and RNA and consist of karyotyping analyses, fluorescence in situ hybridization (FISH), quantitative real-time polymerase chain reaction (RT-PCR), microarray-based comparative genomic hybridization (array CGH) and more recently next-generation sequencing (NGS) [5]. Those techniques enable the detection of chromosomal abnormalities including translocations, recurrent fusion genes, internal chromosomal amplification, and loss or gain of chromosomal region. Those routine techniques used for the clinical diagnosis such as FISH, array CGH or NGS can be laborious, time-consuming and expensive and therefore may be not available or applicable in all research laboratories.

To remedy these limitations, we introduce an optimized method, the Proximity Ligation Assay (PLA), to identify fusion proteins and their cofactors in non-adherent cells that can be easily handled in research field. PLA is accessible to biological laboratories since it does not necessitate specific skills or knowledge and requires common materials found in any molecular and cellular laboratory such as cell culture incubator or epifluorescence microscope.

PLA extends the capabilities of traditional immunoassays and was validated for the first time in 2002 for protein detection [6] and in 2008 for endogenous in situ protein-protein interactions in cell lines [7]**.** PLA enables detection, visualization and quantification of individual endogenous proteins, protein modifications and protein interactions in tissue and cell samples prepared for microscopy. PLA can be performed on many different samples including protein suspensions (e.g. cell lysates), or fixed tissues (e.g. cell culture slides, cytospin preparations or tissue sections). The readout is a fluorescence signal which is easily visualized under a microscope and quantified. This method has many advantages, notably its high sensitivity and specificity, the relatively short duration of the procedure (2 days), the repeatability, and the small number of cells required. Moreover, identifying a fusion protein at the protein level enables evaluation of the expression level of the endogenous fusion protein within the cell, assessment of the protein subcellular localization, and identification of novel protein partners.

For these reasons, we optimize the PLA for detection of fusion proteins and nuclear protein interactions in non-adherent B-cells.

We specifically chose the B-precursor acute lymphoblastic leukemia (B-ALL) as a study model since this disease is the most common childhood malignancy and the leading cause of cancer-related death in children and young adults. The most frequent B-ALL (∼22%) is characterized by the chromosomal translocation t(12;21)(p13;q22) that results in the fusion of two transcription factors, ETV6 and RUNX1*,* producing a functional fusion protein ETV6-RUNX1 previously known as TEL-AML1 [8, 9]. Specifically, we adapted permeabilization buffers and incubation time to investigate the presence of ETV6-RUNX1 fusion protein in pre-B cells, as well as ETV6-RUNX1 interaction with well-known cofactors.

Using this PLA approach, we were able to confirm or deny the existence of the endogenous ETV6-RUNX1 fusion protein in pre-B lymphoblastic cell lines and, more interestingly, in pre-B lymphoblasts from leukemic patients. Additionally, we were able to demonstrate the molecular proximity of CBFB, a well-known cofactor of RUNX1, with ETV6-RUNX1. The optimized PLA procedure can be readily applied to other non-adherent hematological cells, from cell lines or patients’ cells to detect fusion proteins as well as protein interactions.

## Methods

### Cell lines

The REH cell line is a pre-B ALL cell line initiated from the peripheral blood of a patient with pre-B ALL in first relapse [10]. REH cells carry the chromosomal translocation t(12;21) and chromosomal deletion del(12) producing respectively the *ETV6-RUNX1* (previously known as *TEL-AML1*) fusion gene and the deletion of the residual *ETV6* gene (previously known as *TEL*) [11]. The pre-B Nalm6 cell line was also initiated from ALL relapse [12] and presents a near diploid karyotype with a translocation t(5;12)(q33.2; p13.2) [11]. Nalm6 and REH cells were maintained in RPMI 1640 medium (Gibco, Thermo Fisher Scientific) containing 10% heat-inactivated fetal calf serum (Eurobio) supplemented with antibiotics (100 U/mL penicillin-G and 100 U/mL streptomycin, Gibco). The cells were maintained at 37 °C in a humidified incubator under a 5% CO_2_ atmosphere.

### Generation of stable cell lines

Nalm6^shRUNX1^ cells were obtained by transduction of Nalm6 cells in the presence of 4 μg/mL of polybrene (Merck Millipore) with lentivirus bearing MISSION pLKO.1 shRNA-puro vector targeting human RUNX1 (#TRCN0000013660, Sigma-Aldrich).

To obtain stable Nalm6^+RUNX1^ or Nalm6^+ETV6-RUNX1^ cell lines, Halotag-RUNX1 human ORF from pFN21A (#FHC01784, Kazusa collection, Promega), or Halotag-ETV6-RUNX1 (ETV6-RUNX1 ORF subcloned from plasmid kindly provided by G. Nucifora [13]) were cloned into a pLenti CMV-Puro-DEST by Gateway technology. Lenti CMV Puro DEST (w118–1) was a gift from Eric Campeau (Addgene plasmid # 17452) [14]**.** To produce lentivirus, HEK293T cells were co-transfected with pLenti-CMV-Puro-DEST bearing Halotag-RUNX1 or Halotag-ETV6-RUNX1, pSPAX2 and pCMV-VSV-G for packaging using Lipofectamine 3000 Transfection Reagent (Thermo Fisher Scientific). The plasmid psPAX2 was a gift from Didier Trono (Addgene plasmid # 12260) and pCMV-VSV-G was a gift from Bob Weinberg (Addgene plasmid # 8454) [15]. After 48 h, supernatant was harvested, filtered and added to Nalm6 cells with 4 μg/mL polybrene. All the transduced cells were selected in medium containing 0.25 μg/mL puromycin (Invitrogen) as previously established [16].

### Patients’ cells

Bone marrow leukemia cells were collected at diagnosis, after informed consent had been obtained, in accordance with the declaration of Helsinki. The protocol was approved by the ethics committee of Rennes Hospital (Rennes, France). Vital mononuclear cells were isolated from bone marrow by successive centrifugations through lymphocytes separation medium (Eurobio). The detection of chromosomal abnormalities was performed at Rennes University Hospital by FISH analysis.

### Proximity ligation assay (PLA)

PLA was carried out with Duolink® In Situ Detection Reagents Orange (#DUO92007, Sigma Aldrich). Additional reagents Duolink® In Situ PLA® Probe Anti-Rabbit PLUS/MINUS (#DUO92002/DUO92005, Sigma Aldrich) and Duolink® In Situ PLA® Probe Anti-Mouse PLUS/MINUS (#DUO92001/DUO92004, Sigma Aldrich) were used. Optimization of manufacturer’s procedure applied to non-adherent cells is the goal of this article and is described underneath. One representative experiment of at least three independent experiments is shown.

### Antibodies for proximity ligation assay

Duolink™ PLA experiments rely on the selection of two primary antibodies (preferably immunohistochemistry, immunocytochemistry or immunofluorescence validated) that must be raised in two different species (for instance mouse and rabbit). Primary antibodies should be from IgG-class and specific for the target to be detected. PLA can use either monoclonal or polyclonal antibodies. We have tested several antibodies **(**Table 1).

**Table 1:** Antibodies used for PLA assay

Antigen	Species	Name	Concentration	Reference
RUNX1	mouse	Anti-RUNX1	1 mg/mL	ab110035 (Abcam)
RUNX1	rabbit	Anti-RUNX1	1 mg/mL	ab23980 (Abcam)
ETV6	mouse	Anti-ETV6	0.5 mg/mL	ab54705 (Abcam)
ETV6	rabbit	Anti-ETV6	0.2 mg/mL	sc11382 (Santa Cruz biotechnology)
ETV6	rabbit	Anti-ETV6	0.2 mg/mL	sc166865 (Santa Cruz biotechnology)
CBFB	rabbit	Anti-CBFB	1 mg/mL	ab133600 (Abcam)

### Quantitative PCR

RNA was extracted using the NucleoSpin RNA II kit (Macherey Nagel). cDNA was synthesized using High capacity cDNA RT kit (Life Technologies) according to the manufacturer’s protocol. Real-time PCR was carried out in sealed 384-well microtiter plates using the SYBR™ Green PCR Master Mix (Applied Biosystems), according to Applied Biosystems gene amplification specifications (40 cycles of 15 s at 95 °C and 1 min at 60 °C). The following forward (F) and reverse (R) primers were used for RUNX1: F-RUNX1 (5′-ACAAACCCACCGCAAGTC-3′), R-RUNX1 (5′-CATCTAGTTTCTGCCGATGTCTT-3′); and for ETV6-RUNX1: F-ETV6 (5′-AAGCCCATCAACCTCTCTCA-3′), R-RUNX1 (5′-TCGTGGACGTCTCTAGAAGGA-3′). Data analysis was performed using the ΔΔCT-method [17]. The housekeeping genes *GAPDH* or *ABL* were used to normalize the data. The log2 fold change of all genes of interest was calculated compared to Nalm6 control cells.

### Western blot analysis

RUNX1 and ETV6-RUNX1 proteins were detected via immunoblot using an anti-RUNX1 mouse antibody (#110035, clone 5A1, Abcam) diluted at 1:300. ETV6 proteins were detected via immunoblot using an anti-ETV6 mouse antibody (#54705, Abcam) diluted at 1:1000. As a loading control, HSC70 protein levels were assessed using a mouse-derived antibody (#7298, clone B-6, Santa Cruz) diluted at 1:500. The immunoblots were visualized with enhanced chemiluminescence Western blotting detection system (WBKLS0500, Merck Millipore) according to the manufacturer’s instructions.

## Method for the optimized proximity ligation assay (PLA) for non-adherent pre-B lymphoblasts

### Principle

PLA technology allows the detection of interactions between endogenous proteins. This technique is based on the detection of protein proximity. PLA uses one pair of primary antibodies. Those primary antibodies target proteins of interest, for instance two epitopes of a fusion protein, or two distinct proteins for which we want to study the proximity. Primary antibodies are raised in different species and are detected with secondary antibodies conjugated to short DNA oligonucleotides. If the oligonucleotides are in close proximity (theoretically less than 40 nm) the DNA strands hybridize and participate in rolling circle DNA synthesis. These DNA copies can further be detected through hybridization of fluorescent-labeled oligonucleotides. The resulting high concentration of fluorescence is easily visualized under a microscope and quantified [18]. One dot corresponds to one colocalization.

**Fig. 1:**
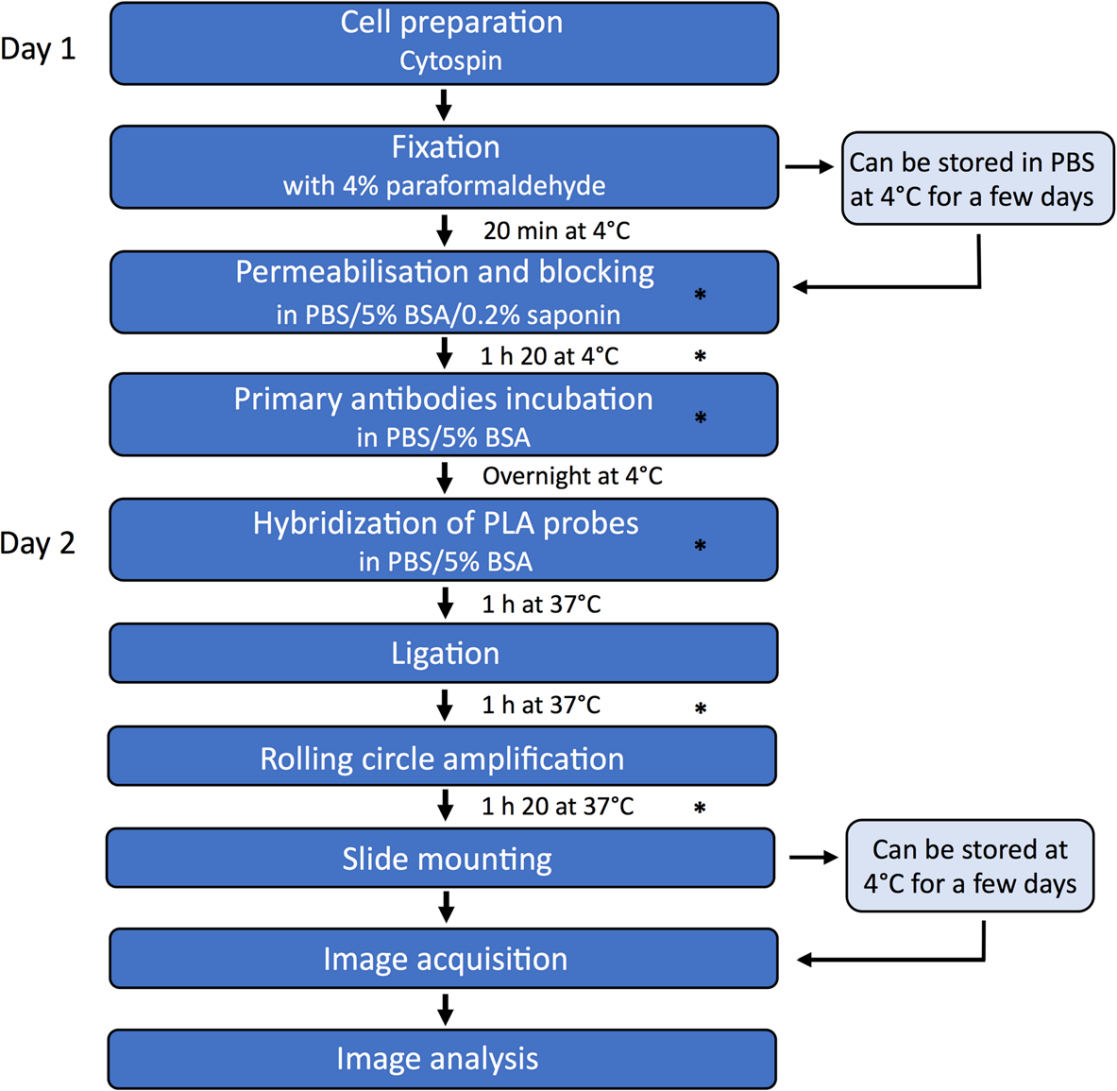
Optimized protocol outlines of Proximity Ligation Assay for non-adherent pre-B lymphoblasts. Schematic outline summarizing the procedure of PLA for detection, visualization and quantification of individual endogenous proteins, protein modifications and protein interactions. Asterisk depicts steps that have been adapted from the original manufacturer’s procedure

Because PLA can detect endogenous proteins, and requires few cells, we decided to broaden PLA technology to include the identification of fusion proteins and protein interactions in hematological malignant cells. For that purpose, we chose to optimize the detection of ETV6-RUNX1 fusion protein and the interaction between RUNX1 and CBFB, its well-known cofactor, in non-adherent pre-B lymphoblasts. A flowchart of the successive steps of the optimized PLA protocol is presented in Fig. 1. Briefly, the cells are cytospun, fixed, permeabilized, and incubated with two primary antibodies raised in different species. Then, we performed hybridization with PLA probes, ligation of the probes, rolling circle amplification, slide mounting, image acquisition by microscopy and image analysis (Fig. 1).

### Cell preparation, fixation and permeabilization

Non-adherent pre-B Nalm6 cells, REH cells and mononuclear cells from human bone marrow were collected by centrifugation. The cells were washed in cold 1X Phosphate Buffer Saline (PBS) (#ET330, Euromedex) and diluted to reach the concentration of 320,000 cells/mL. The Superfrost™ Menzel-Glaser microscope slides (#10143560 W90, Thermo Fisher Scientific) and disposable sample chambers (Shandon™ Cytofunnel™ double, 28mm^2^, #5991039, Thermo Fisher) were placed into appropriate slots in the Shandon Cytospin® 4 Cytocentrifuge (Thermo Scientific). Then, 250 μL of cell suspension were aliquoted in each chamber to drop approximately 80,000 cells per spot. The optimal cellular confluence for PLA experiments is 40–70% confluency after cytospin. The cytocentrifugation was run at 800 rpm during 5 min under low-acceleration. Then, chambers were removed and the areas with cells were encircled using a hydrophobic delimiting pen (Dako pen, #S200230–2, Agilent). The samples were fixed with 20 μL of 4% paraformaldehyde for 20 min at 4 °C and were washed twice in 1X PBS for 5 min in a staining jar (spots must be well covered) under shaking (60–90 rpm/min). We typically used a 70 mL staining jar for 5 slides. After fixation, the samples should not be left to dry at any case before the final step. The cells were blocked and permeabilized by adding 5% Bovine Serum Albumin Fraction V (BSA, #10735094001, Roche) and 0.2% saponin (#47036, Sigma Aldrich) in 1X PBS for 1 h 20 at 4 °C in a humidity chamber. A washing step was finally performed before incubation with primary antibodies in PBS/0.2% saponin for 5 min under shaking at room temperature in a staining jar.

### Preparation of the antibodies

According to the manufacturer’s instructions, primary antibodies should be from IgG-class, specific for the target to be detected and preferably affinity purified. The primary antibodies can be either polyclonal or monoclonal. To maximize the specificity, antibodies should be validated for immunohistochemistry, and/or immunofluorescence. When using two primary antibodies targeting the same protein, they must be directed against different, non-competing epitopes. The two primary antibodies must have been raised in different species. Moreover, both primary antibodies must bind to the target under the same conditions (fixation, buffer etc.).

Antibodies used in this protocol are presented in Table 1. Primary antibodies were added at 0.1 mg/mL final in 15 μL preblocking buffer (PBS with 5% BSA) and incubated at 4 °C overnight into a humidity chamber to prevent evaporation. The droplet must cover the reaction area. Typically, for 0.28 cm^2^, it is not recommended to use less than 15 μL of total reaction volume on the spot.

The next day, the slides were washed three times 5 min each in a staining jar under gentle shaking (60–90 rpm/min) at room temperature: first with 1X PBS/0.5% tween-20 (#P9416, Sigma Aldrich), second with 1X PBS/0.2% saponin/0.5% tween-20 and the third with 1X PBS/0.5% tween-20. Then, secondary antibodies conjugated with oligonucleotides (PLA probe anti-species 1 MINUS and PLA probe anti-species 2 PLUS) were diluted 1:5 in preblocking buffer and 15 μL/spot was applied for 1 h at 37 °C in a humidity chamber.

### Ligation and amplification (Duolink in situ detection kit, Sigma Aldrich)

The wash buffers should be brought to room temperature before use, as low temperature slows down the enzymatic reactions. The slides were washed four times, 5 min each in a staining jar under gentle shaking (60–90 rpm/min) at room temperature: first with 1X PBS/0.5% tween-20, second with 1X PBS/0.2% saponin/0.5% tween-20, third with 1X PBS/0.5% tween-20, and fourth with 1X PBS. The 5X Ligation buffer was diluted in water for a final concentration of 1X and the ligase (1 U/μL) diluted in 1:40 in the ligation 1X solution. Fresh dilutions should be prepared just before use. Samples were incubated with 15 μL of ligase solution for 1 h at 37 °C in the humidity chamber.

Then, the slides were washed three times, 5 min each in a staining jar under gentle shaking (60–90 rpm/min) at room temperature: 1X PBS/0.2% saponin/0.5% tween-20, second with 1X PBS/0.5% tween-20 and the third with 1X PBS. The amplification 5X was diluted extemporaneously in water for a final concentration of 1X and the polymerase (10 U/μL) diluted in 1:80 in the amplification 1X solution. Samples were incubated with 15 μL of amplification solution for 1 h 15 at 37 °C in the humidity chamber. The samples should be protected from light in order to avoid bleaching of the fluorophores.

### Final wash and mounting

The slides were washed three times 5 min each in a staining jar under gentle shaking (60–90 rpm/min) at room temperature: first 1X PBS/0.2% saponin/0.5% tween-20, second with 1X PBS/0.5% tween-20 and the third with 1X PBS. The outline of circles was dried and slides were mounted with a cover slip using a minimal volume of Mounting Medium with DAPI (#H-1200, Clinisciences). The slide was then sealed with nail polish and analyzed on an epifluorescence microscope.

### Fluorescence imaging

Images of the cells were acquired under a DeltaVision Elite High Resolution epifluorescence microscope, using appropriate filters. The fluorophore for the Amplification Orange kit has an excitation wavelength of 554 nm and an emission wavelength of 579 nm and can be detected using the TRITC filter. A DAPI filter, excitation 360 nm and emission 460 nm, was used for the nuclear staining. Images were captured by a photometrics cool snap HQ2 camera utilizing the image capture software softWoRx version 5.5. Slides were analyzed under a 20X/0.85 oil magnification objective.

During image capture, 5–7 images were taken on each spot. The exposure time was set so that PLA signals were easily distinguishable but not overexposed. It varied between 200 and 500 ms for TRITC with 10% exposure and 200–400 ms for DAPI with 5% exposure. Each image was captured on one layer in the focus plane (DAPI). For TRITC filter, few signals can be visualized either above or below the current focus but the low thickness of the cells after the cytospin allowed all dots to be visible in one focus. If necessary, to increase the resolution, 8 layers (separated for instance by 0.3 nm each) can be taken and a "Z-projection" on the maximum intensity can be applied on the picture. The captured images can be saved as .dv or .tiff.

### Image analysis

With the PLA approach, since each dot represents a high concentration of fluorescence (several hundred–fold replication of the DNA circle) as a result of the probe proximity, the dot number can be quantified independently of the intensity. The fluorescent particle analysis was performed and automated with ImageJ software (https://imagej.nih.gov/ij/). The script for .dv images (DeltaVision) is presented in Table 2. The two channels (for instance DAPI and TRITC) were separated to analyze the image from DAPI-nuclear staining separately from the image of the TRITC-channel associated to the PLA dots. First, on the DAPI-channel image, the filter "Smooth" surface was applied to the image. Then, the best threshold was set to identify nucleus and to convert the image to binary (black and white) image. To help separating touching nuclei, morphological functions were processed as followed: "Close" command (*i.e.* a "Dilation" operation followed by "Erosion", to fill nuclei) and "Open" command (*i.e. * "Erosion" operation followed by "Dilation", to smooth the image and remove isolated pixels). The overlapping nuclei were separated using the "Watershed" function. The "Analyze particles" command was used to count the number of separated nuclei on the image, and results were added to ROI (region of interest) manager. In this step, the appropriate minimum and maximum pixel area sizes was set and cells on picture edge were excluded.

**Table 2:** ImageJ SCRIPT for .dv image (DeltaVision format) analysis

STEPS	SCRIPT
1. Separate the different channels (C1- for DAPI-nuclear staining and C2- for TRITC-channel associated to the PLA dots)	- imageName = getTitle();
	- run (“Split Channels”);
	- selectWindow (“C1-” + imageName);
	- selectWindow (“C2-” + imageName);
	- selectWindow (“C1-” + imageName);
	- run (“Smooth”);
	- run (“Median...”, “radius = 2”);
2. Apply threshold on C1	- setAutoThreshold (“Default dark”);
	- //run (“Threshold...”);
	- setOption (“BlackBackground”, false);
	- run (“Convert to Mask”);
3. Apply morphological filters on C1	- selectWindow (“C1-” + imageName);
	- run (“Close-”);
	- run (“Open”);
4. Segment nuclei on C1	- run (“Watershed”);
	- run (“Sharpen”);
	- run (“Clear Results”);
5. Count individual nuclear staining on C1	- run (“Analyze Particles...”, “size = 75–550 show = [Overlay Outlines] exclude clear add”);
6. Count individual PLA dots on C2	- selectWindow (“C2-” + imageName);
	- run (“Find Maxima...”, “noise = 60 output = [Single Points] exclude”);
	- selectWindow (“C2-” + imageName + “Maxima”);
7. Divide by 255 on the “find maxima output”	- run (“Divide...”, “value = 255”);
	- run (“Set Measurements...”, “area integrated redirect = None decimal = 3”);
8. Measure the pixel values	- roiManager (“Measure”);
9. Get the results of the number of PLA dots in each nucleus	- String.copyResults();
	- selectWindow (“C2-” + imageName + “Maxima”);
	- close();

Second, on the FITC-channel image, the "Find maxima" function was applied. The noise tolerance has to be determined initially (depending on the picture resolution), and the output type selected was "Single point". The number of dots in each nucleus was calculated with the "Measure" command from the ROI manager, to allow all regions previously identified to be represented on the single point output image. In the results window, the raw integrated density (RawIntDen) represents the sum of pixel values in each nuclear staining. Because the single point output is in black and white and because we had 8-bit images (28 equals to 256 different pixel values, in the range 0–255), we divided the RawIntDen values by 255 to obtain the number of detected maxima/dots in each region defined by the nuclear stain.

### Statistical analysis

Statistical analyses were performed with GraphPad Prism 6.0 software. Mean values ± standard deviation (S.D.) are presented. The assay cut-off value is set to two standard deviations over the background signal according to Nordengrahn et al. [19]. The background signal is estimated with a pair of antibodies that is known to not interact. Samples with values below this cut-off are considered to be negative for the interaction of interest while samples with values higher than the threshold are positive.

## Results

### Validation of optimized PLA on pre-B lymphoblast cell lines for detection of proximity between two endogenous proteins

**Fig. 2:**
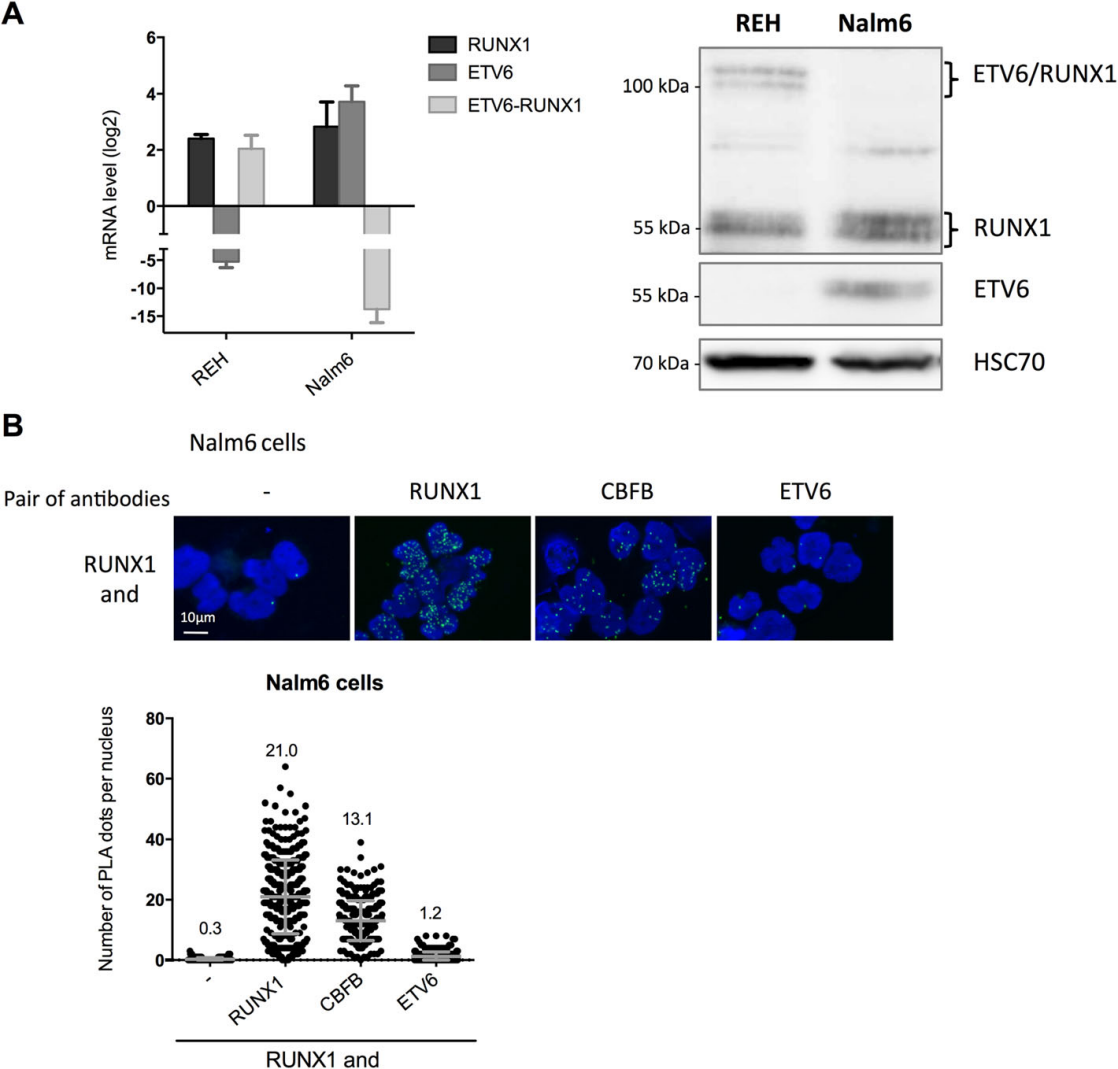
Validation of PLA on pre-B lymphoblasts. **a** Quantitative real-time RT-PCR and western blot analysis showed respectively mRNA and protein expression of RUNX1, ETV6 or ETV6-RUNX1 in Nalm6 and REH cells. All RT-PCR (left panel) were performed in triplicate and gene expression was normalized to *ABL1* expression (error bars are S.D.) while western blot analyses (right panel) are representative images from the whole-cell lysates. **b** Technical controls demonstrate the specificity of PLA signals in Nalm6 cells and the proximity between two proteins (RUNX1 and CBFB). Nuclei were stained with DAPI. RUNX1 ab110035 antibodies were incubated alone (−) or with RUNX1 ab23980, CBFB ab133600 or ETV6 sc11382 antibodies. Each picture (upper panel) is representative of a typical cell staining observed in 5 fields randomly chosen. The quantification of the number of PLA dot per nucleus is presented with the mean values ± S.D

We first performed Proximity Ligation Assay (PLA) on Nalm6 lymphoblasts. Those cells express both RUNX1 and ETV6 transcripts and proteins [12] (Fig. 2a). Specificity of the assay was shown by lack of non-specific staining in the negative control, displayed by the use of mouse-RUNX1 antibody alone (Fig. 2b). On the contrary, incubation with two different-species-antibodies directed against RUNX1 showed on average 21.0 (±12.2, *n* = 425) fluorescent dots per cell validating the presence of RUNX1 proteins in Nalm6 cells. As expected, RUNX1 proteins were localized in the nuclei. Detection of RUNX1 and CBFB proximity showed on average 13.1 (±6.6, *n* = 299) dots per cell. This colocalization between RUNX1 and CBFB is in concordance with the literature [20, 21]. We next wanted to assess the proximity of the proteins RUNX1 and ETV6. PLA with anti-mouse RUNX1 antibodies and anti-rabbit ETV6 antibodies did not show any relevant interaction (about 1.2 ± 1.5 dot per nucleus, *n* = 366) in Nalm6 cells. This result is consistent with the literature and expression results; we did not expect to detect interactions between endogenous ETV6 and RUNX1 proteins in Nalm6 cells. According to this result, the cut-off assigned in this study was a mean of 4.2 PLA dots per nucleus (two standard deviations over the background signal [19]), representing the fluorescent background or the probability of the 2 proteins to be in a close proximity by chance.

Our results show that our optimized PLA protocol is selective and suitable for detection of molecular proximity between two distinct proteins in non-adherent cells. Here, we validated the efficiency of the optimized procedure to detect the well-known protein interaction between the protein RUNX1 and its canonical molecular co-factor CBFB in Nalm6 pre-B lymphoblasts.

### Sensitivity of optimized PLA on non-adherent cells

**Fig. 3:**
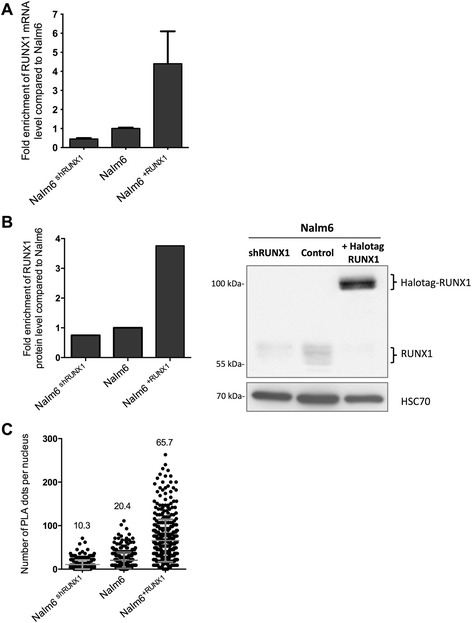
PLA is sensitive to total protein level. **a** Nalm6 wild type, depleted for RUNX1 protein (Nalm6^shRUNX1^) or overexpressing RUNX1 (Nalm6^+RUNX1^) cell lines were validated using RT-qPCR. Results are presented in terms of a fold change after normalizing *RUNX1* mRNA levels with *GAPDH* mRNA. Each value represents the mean of ± S.D. of three independent transduced cells. **b** Representative images of western blot (*left* panel) and densitometry analysis (*right* panel) showing the quantification of RUNX1 protein level normalized to HSC70  **c** Quantification of the PLA signal (dots plots) on Nalm6 cells displaying a gradient of expression of RUNX1 is represented. Nalm6, Nalm6^shRUNX1^ or Nalm6^+RUNX1^ cells were incubated with a pair of RUNX1 antibodies (the mean values ± S.D. are presented)

To evaluate the sensitivity of the assay, we performed PLA on Nalm6 cells displaying a gradient of expression of RUNX1. We generated Nalm6 cell lines depleted for RUNX1 transcript and protein (Nalm6^shRUNX1^) or overexpressing RUNX1 (Nalm6^+RUNX1^) (Fig. 3a and 3b). Quantification of RUNX1 protein level by PLA positively correlated with quantification of RUNX1 protein level demonstrated by western blot (Fig. 3b and 3c). This result demonstrates that PLA may be sensitive to protein level.

### Detection of overexpressed and endogenous fusion protein by PLA

**Fig. 4:**
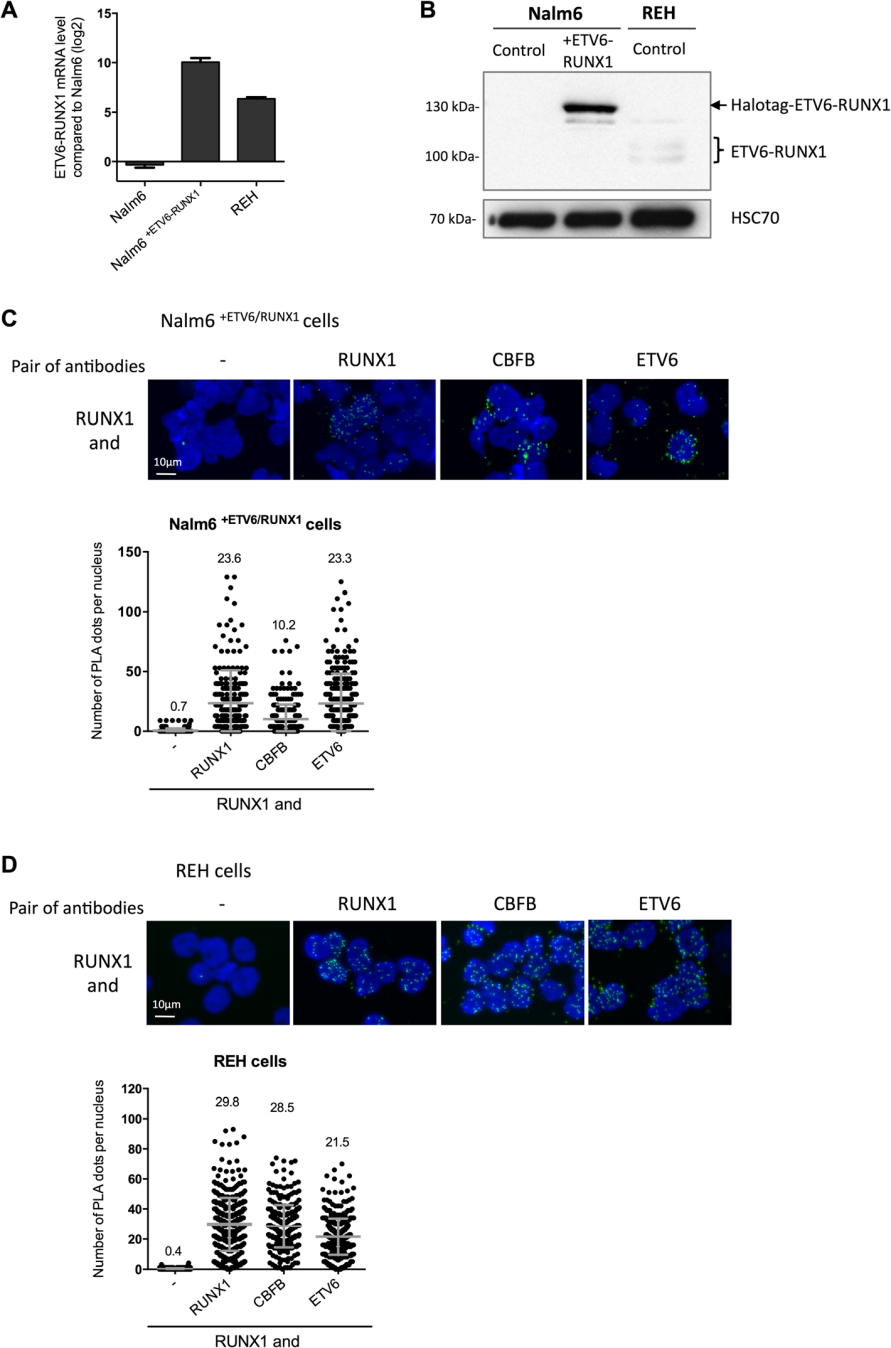
Our optimized PLA protocol effectively detects overexpressed or endogenous fusion proteins in pre-B lymphoblasts. The Nalm6 cell line overexpressing ETV6-RUNX1 (Nalm6^+ETV6-RUNX1^) was validated using RT-qPCR (**a**) and western blot (**b**). **a** Results are presented in terms of a fold change after normalizing *ETV6-RUNX1* mRNA levels with *GAPDH* mRNA. Each value represents the mean of ± S.D. of three independent transduced cells. **b** Representative images of western blot showing expression of RUNX1 or HSC70 proteins in both cell lines are represented. **c** Pictures and quantification of PLA signals on Nalm6 cells overexpressing ETV6-RUNX1 protein (Nalm6^+ETV6-RUNX1^ cells) (**a**) or on REH cells that expressed endogenous ETV6-RUNX1 protein (**b**). Nuclei were stained with DAPI. RUNX1 ab110035 antibodies were incubated alone (−) or with RUNX1 ab23980, CBFB ab133600 or ETV6 sc11382 antibodies. Each picture (upper panel) is representative of a typical cell staining observed in 5 fields chosen at random. The quantification of the number of PLA dots per nucleus is presented with the mean values ± S.D

Having proven the selectivity and the sensitivity of the optimized PLA protocol, we next addressed the question of the detectability of fusion proteins (*i.e.* ETV6-RUNX1) using our optimized PLA protocol. To that aim, we overexpressed ETV6-RUNX1 transcript and protein (Fig. 4a and 4b) in Nalm6 cells (Nalm6^+ETV6-RUNX1^ cells) and carried out PLA (Fig. 4c). The negative control (RUNX1 antibodies alone) displayed 0.7 (± 1.8, *n* = 296) dots demonstrating a very low background. The positive control (detection of RUNX1 protein using two anti-RUNX1 antibodies) showed 23.6 (± 27.6, *n* = 302) dots per nucleus. In those Nalm6^+ETV6-RUNX1 ^ cells, protein proximity between RUNX1 and CBFB was maintained (10.2 dots per nucleus) as observed in Nalm6 cells. Detection of ETV6-RUNX1 fusion protein showed 23.3 (± 25.0, *n* = 419) dots per nucleus in Nalm6^+ETV6-RUNX1^ cells whilst the same mix of antibodies detected less than 1.2 dots per cells in Nalm6 cells (Fig. 2b and 4c). This result convincingly demonstrates that PLA can be a powerful tool to detect overexpressed fusion proteins. We wanted further to detect the endogenous ETV6-RUNX1 fusion protein. For that purpose, we used REH cells which harbor the chromosomal translocation t(12;21)(p13;q22) that generates the fusion gene and protein ETV6-RUNX1 (Fig. 2a). PLA using single-species RUNX1 antibodies showed no background (0.4 dots per nucleus; negative control) whereas incubation with two species-different antibodies directed against RUNX1 displayed on average 29.8 dots (± 17.6, *n* = 426) (positive control) (Fig. 4d) confirming the efficiency of the protocol on REH cells. Importantly, PLA using a mix of anti-RUNX1 and anti-ETV6 antibodies showed 21.6 ± 12.0 (*n* = 415) dots per cells. We observed that the majority of ETV6 and RUNX1 proximities were localized within the nucleus. Because the non-translocated allele of ETV6 is absent in REH cells, this result of ETV6 and RUNX1 proximity means the presence of ETV6-RUNX1 fusion protein in those cells. We were next interested in assaying the molecular proximity between the ETV6-RUNX1 fusion protein and CBFB. To the best of our knowledge, even if this interaction was suspected [22], it has never been formally demonstrated so far. PLA using a mix of anti-CBFB and anti-RUNX1 antibodies, as well as PLA using a mix of anti-CBFB and anti-ETV6 antibodies revealed a molecular proximity between ETV6-RUNX1 and CBFB (Fig. 4d). Those data demonstrate that PLA can be a powerful tool to detect overexpressed as well as endogenous fusion proteins in cell lines.

### Validation of PLA antibodies pairs using detection of fusion protein

Various antibodies against ETV6 and RUNX1 are available. Three different pairs of antibodies against ETV6 and RUNX1 were tested in REH cells expressing the ETV6-RUNX1 fusion protein to define the most suitable couple for the assay in patients (Additional file 1: Figure S1). The couple anti-mouse RUNX1 with anti-rabbit ETV6 is the most effective and will be used for ETV6-RUNX1 detection in patients.

### Identification of ETV6-RUNX1 fusion protein on patient lymphoblast cells

Given the high selectivity and sensitivity of our optimized PLA protocol for detecting close proximity between two proteins and validating the presence of a fusion protein, we wondered whether it was beneficial to use this protocol on patient cells to confirm the presence of the fusion protein suspected by the identification of a t(12;21)(p13;q22) chromosomal translocation.

To that aim, PLA was performed on samples of mononuclear cells from 4 patients with pre-B-ALL suspicion. Karyotype features and fusion transcript of the patients were presented in Table 3. The cohort was composed of 3 males and 1 female with a median age of 4-year-old. Lymphoblasts from two of them (patients #3 and #4) were positive for t(12;21) chromosomal translocation and ETV6-RUNX1 fusion transcript while the two others patients’ lymphoblasts (patients #1 and #2) did not carry this translocation. PLA was carried out on an aliquot of the bone marrow diagnosis sample. As previously, we used a single antibody anti-RUNX1 as negative control, and two antibodies against RUNX1 as positive control.

**Table 3:** Biological and cytogenetic characteristics of the B-acute lymphoblastic patients

Patient	Sex	Age at diagnosis (year)	Cytogenetic	Fusion transcript
#1	Male	2	Hyperploidy 3 copy RUNX1 2 copy ETV6	none
#2	Female	8	t(1;19)	E2A-PBX1
#3	Male	4	t(12;21) del ETV6	ETV6-RUNX1
#4	Male	3	t(12;21) del ETV6	ETV6-RUNX1

**Fig. 5:**
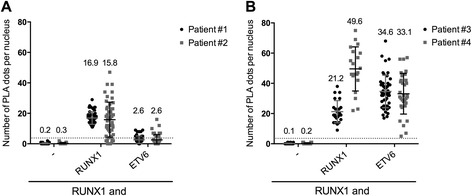
Proximity Ligation Assay is a reliable tool for the detection of ETV6-RUNX1 fusion protein in patients’ lymphoblasts. Quantification of PLA signals per nucleus in lymphoblasts from two patients negative (**a**) or positive (**b**) for the fusion protein ETV6-RUNX1. The cells were incubated with RUNX1 ab110035 antibodies alone (−) or with RUNX1 ab23980, CBFB ab133600 or ETV6 sc11382 antibodies. The quantification of the number of PLA dot per nucleus is presented with the mean values ± S.D. The line represents the cut-off used to determine positive PLA signals

For patient #1 and patient #2 (Fig. 5a), the negative controls showed no dots, confirming the high specificity of the procedure. The positive controls showed about 16.9 dots per nucleus (±4.1, *n* = 50) for patient #1, and 15.8 (±11.5, *n* = 50) for patient #2. PLA with a mix of anti-RUNX1 and anti-ETV6 showed about 2.6 (±2.4, *n* = 50) for patient #1 and 2.6 (±3.5, *n* = 48) dots per nucleus for patient #2. Therefore, the mean number of dots per nucleus for ETV6 and RUNX1 proximity is below the cut-off level for both patients. We concluded that both patient #1 and patient #2 lymphoblasts did not express the fusion protein ETV6-RUNX1, which were concordant with FISH analyses

For patient #3 (Fig. 5b), the controls produced expected results: no dots with the negative control (anti-RUNX1 alone) and 21.2 (±7.3, *n* = 24) dots per nucleus with the positive control (2 different anti-RUNX1 antibodies). Interestingly, the mix of anti-RUNX1 and anti-ETV6 revealed 34.6 PLA dots (34.6 ± 10.8, *n* = 49) per nucleus, demonstrating a close proximity between ETV6 and RUNX1 that we allocated to the presence of the ETV6-RUNX1 fusion protein. The presence of the fusion protein was validated by the FISH result later on. Similarly, in patient #4 (Fig. 5b), the non-specific background was negative and the pair of anti-RUNX1 gave a positive result with 49.6 (±14.6, *n* = 20) dots per cell. PLA with the mix of anti-RUNX1 and anti-ETV6 showed an equivalent level of detected interactions, 33.1 (±13.5, *n* = 32) dots per nucleus that we attributed to the presence of the fusion protein ETV6-RUNX1. The FISH result was concordant with our PLA result. We conclude that the optimized PLA can also be used on non-adherent cells from patients, facilitating the study of fusion protein and protein-protein interactions and their subcellular localization.

## Discussion

The purpose of the current study was to develop an optimized PLA protocol for non-adherent cells using commercially available materials and kits, and common molecular and cellular biology laboratory materials. By taking the example of ETV6-RUNX1 fusion protein, we present an improved method for the detection of fusion proteins and their partners that can be an important tool in scientific and translational research. PLA is an innovative method of protein-protein colocalization detection by molecular biology that combines the advantages of microscopy, specifically the requirement of few cells and in situ visualization, with the advantages of molecular biology precision, enabling detection of protein proximity theoretically ranging from 0 to 40 nm. We provide compelling negative and positive controls, and demonstrate that the optimized PLA procedure is sensitive to total protein level.

We overcome the issue of maintaining non-adherent hematological cells by using cytocentrifugation, and optimized buffers, incubation times, and washing steps. Because hematological cells are more resistant to permeabilization and to preserve protein integrity, we increased permeabilization time to 1 h 20 at 4°C, instead of 30 min at 37°C as recommended by the manufacturer. We adapted the primary antibody buffer to our cells. We increased all washing times and the stringency of buffers to reduce non-specific fluorescence. We increased the ligation incubation time from 30 min to 1 h. Finally, we optimized the amplification step (80 min at 37 °C instead of 100 min) to reduce the potential coalescent signals.

As expected, the specificity of the assay depends on using an appropriate pair of antobodies. The pair of antibodies works in conjunction, meaning that specificity and efficiency is limited by the least specific and efficient antibody. Therefore, excellent antibody quality is an important parameter for this method. We strongly recommend validating antibody specificity and efficiency. Validation of antibodies by immunofluorescence is also highly recommended. We have also validated pairs of antibodies by PLA. PLA can be performed in a single recognition experiment using different primary antibodies against the same protein. The couple showing the smaller number of dots is supposed to contain at least one limiting antibody that may be avoided for PLA assay.

As the PLA technique is very sensitive, special care is needed to keep the incubation times and conditions equal for different samples. In addition, we draw attention on the fact that the number of dots may vary from one experiment to another. Obviously, the duration of each step is crucial and should be strictly respected. We have also observed a slight decrease of efficiency of the kit over time. To prevent enzyme activity degradation, enzymes must be kept at −20 °C and added just before applying the reaction mixture to the sample. To overcome the kit limitation, we also recommend the systematic inclusion of a positive control by a single protein recognition (e.g. detection of total RUNX1 using 2 RUNX1 antibodies) for each experiment. This positive control can be used to normalize PLA data, allowing comparison between different series of experiments.

The total workflow lasts 2 days. This duration accepts some variation. Cells can be stored for a few days in PBS after fixation at 4 °C. We recommend using fresh cells whenever possible but cryopreserved cells in fetal calf serum/10% DMSO could also be used. Moreover, before image acquisition, the stained cells can be kept for a few days in the dark at 4 °C before the fluorescent signal decreases.

The optimized PLA protocol has been achieved on pre-B lymphoblast cells, and validated also on other non-adherent cells such as hematopoietic stem cells, multipotent progenitors, progenitors of granulocytes and macrophages and multi-lymphoid progenitors (data not shown); demonstrating that the optimized protocol is a robust assay for non-adherent cells. Therefore, our optimized PLA protocol resolves both selectivity and sensitivity issues in hematological non-adherent cells [23].

Numerous disorders are characterized by abnormal protein subcellular localizations, interactions or fusion proteins. Our optimized PLA protocol allows visualization of protein localization within the cells.

## Conclusion

Our experiments demonstrate that the optimized PLA protocol enables molecular and cellular biologists to detect fusion proteins, subcellular expression, and protein interactions in non-adherent cells, and therefore provides a new tool for leukemia pathogenesis research. In conclusion, the optimized proximity ligation assay for non-adherent cells described here, is simple, fast, reliable, and can be adopted for a wide range of applications in the biological field.

## Additional file

Additional file 1: Figure S1. PLA with three different pairs of antibodies against ETV6 and RUNX1. Quantification of PLA signals per nucleus in REH cells. Three different pairs of antibodies against ETV6 and RUNX1 were tested as indicated in the figure. (PDF 77 kb)

## Abbreviations

Array CGH: Microarray-based comparative genomic hybridization
B-ALL: B-precursor acute lymphoblastic leukemia
BSA: Bovine serum albumin
FISH: Fluorescence in situ hybridization
NGS: Next-generation sequencing
PBS: Phosphate buffered saline
PLA: Proximity ligation assay
RawIntDen: Raw integrated density
RT-PCR: Quantitative real-time polymerase chain reaction
S.D.: Standard deviation
