# Supplementary material for: Optimization of proximity ligation assay (PLA) for detection of protein interactions and fusion proteins in non-adherent cells: application to pre-B lymphocytes
Source: Mol Cytogenet. 2017 Jul 20;10:27. doi: 10.1186/s13039-017-0328-2 (PMC5520345; doi:10.1186/s13039-017-0328-2)
Supplement: Additional file 1: Figure S1. — PLA with three different pairs of antibodies against ETV6 and RUNX1. Quantification of PLA signals per nucleus in REH cells. Three different pairs of antibodies against ETV6 and RUNX1 were tested as indicated in the figure. (PDF 77 kb) [file 13039_2017_328_MOESM1_ESM.pdf]

Supplemental figure 1

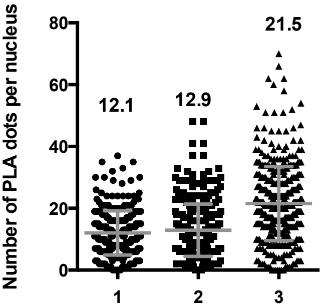

Mouse and rabbit antibodies:

- 1: ETV6 ab54705 and RUNX1 ab23980
- 2: ETV6 sc166835 and RUNX1 ab23980
- 3: RUNX1 ab110035 and ETV6 sc11382
